# Accuracy of 3D-navigated screw fixation in pelvic ring fractures: a single-centre consecutive observational case series

**DOI:** 10.1007/s00590-025-04541-9

**Published:** 2025-10-10

**Authors:** R.A. Timmer, P. van der Zwaal, S.A.G. Meylaerts

**Affiliations:** 1 Department of Trauma Surgery, Leiden University Medical Center, Leiden, Netherlands; 2 Department of Trauma Surgery, Haaglanden Medical Center, The Hague, Netherlands

**Keywords:** Pelvic ring fractures, Screw fixation, 3D navigation, Safety

## Abstract

**Purpose:**

Conventional 2D fluoroscopy-based screw fixation of pelvic ring fractures has high screw misplacement rates and can be technically challenging. Implementation of 3D image guidance is presumed to simplify screw placement and improve procedural safety. The objective of this study was to evaluate the accuracy of 3D navigation in percutaneous screw fixation of pelvic ring fractures.

**Methods:**

A consecutive case series including all patients undergoing 3D-guided screw fixation of pelvic ring fractures between 2019 and 2022 was conducted. Primary study endpoints were screw misplacement, neurological complications, and surgical site infections. Data were analysed using descriptive statistics.

**Results:**

A total of 90 consecutive patients (180 screws) were included, with 120 transsacral-transiliac screws (TSTI) and 60 sacral-iliac (SI) screws used for posterior fixation, 5 retrograde transpubic screws placed for anterior fixation, and 23 antegrade screws placed into the superior rami. For two patients (2.7%), screw misplacements were observed. No neurological complications occurred due to screw misplacement.

**Conclusions:**

The result of the current study shows that 3D navigation provides excellent screw placement accuracy, with minimal screw misplacements and no neurological complications due to screw misplacement.

## Purpose

Over the last decade, the interest and use of minimally invasive techniques in orthopaedic-trauma surgery has increased, since it is associated with less post-operative pain, shorter hospital stays, and fewer complications[1]. When it comes to percutaneous screw fixation of pelvic ring fractures, screw misplacement is frequently reported, with conventional 2D fluoroscopy resulting in screw misplacements of up to 20% [2, 3]. In particular, sacral-iliac (SI) or transsacral-transiliac screw (TSTIS) fixation using 2D fluoroscopy is challenging due to complex three-dimensional morphology of the sacrum. The anatomy of the sacrum is characterized by narrow osseous corridors and the close proximity of neurovascular structures, with sacral dysmorphism being a frequent additional difficulty. [4–6]. Additional factors, such as bowel gas, impaired visualization in obese patients, or decreased bone density in geriatric patients, can further reduce intraoperative visibility and increase the risk of iatrogenic neurovascular injury.[7, 8].

Despite the high incidence of screw misplacement and its potentially devastating effects, conventional 2D fluoroscopy, as first described by Matta and Saucedo in 1989, remains the widely accepted standard technique [9, 10]. In recent years, the use of peri-operative 3D imaging and navigation has increased exponentially. In spine surgery, there is already a clear consensus on 3D-navigated pedicle screw placement, demonstrating significant reduction in screw misplacement, operation time, and radiation dose [11, 12]. These advantages suggest that similar benefits could be achieved in pelvic fracture surgery.

3D-navigated screw placement is rapidly growing in popularity because of its potential superior accuracy compared to 2D-navigated screw placement in pelvic ring fractures [13]. Therefore, the primary goal of this consecutive case series is to determine the accuracy of 3D-navigated screw placement in pelvic ring fractures but also include the potential complications including neurovascular complications, surgical site infections, operation duration, and radiation exposure.

## Methods

### Patient selection and data collection

All consecutive patients with pelvic ring fractures who underwent 3D-navigated screw fixation at an urban Level 1 trauma centre in the Netherlands between 2019 and 2022 were included in this observational case series. Patients who underwent open reduction and internal fixation were excluded.

Baseline characteristics (patient demographics, trauma mechanism, fracture classification) and clinical timepoints (admission, surgery, discharge) were systematically recorded for all included patients. For elderly patients (> 70 years), fracture classification was determined using both the Young and Burgess classification and the classification proposed by Rommens et al. [14, 15].

For all patients, outcomes were analysed in four categories: technical, surgical, neurological, and material-related complications. Technical complications were defined intraoperative K-wire and/or screw misplacement, i.e. perforation of the cortical bone (foraminal or anterior sacral cortex). Surgical complications included post-operative surgical site infections (SSIs), subdivided into superficial infections treated with antibiotics and deep infections requiring operative debridement. Neurological complications included loss of sensation, decreased motor function, and persistent neuropathic pain. For all patients experiencing neurological complications after surgery, a neurologist was consulted to differentiate between pre-existing, trauma-induced, or surgery-related deficits. Material-related complications included screw breakage, backing out of screws, and persistent pain attributed to the implants. Results regarding the radiation dose, effective dose (millisievert, mSv), and dose length product (DLP, Gy·cm^2^) were obtained from the O-arm’s built-in dosimeter.

### Surgical procedure

**Fig. 1:**
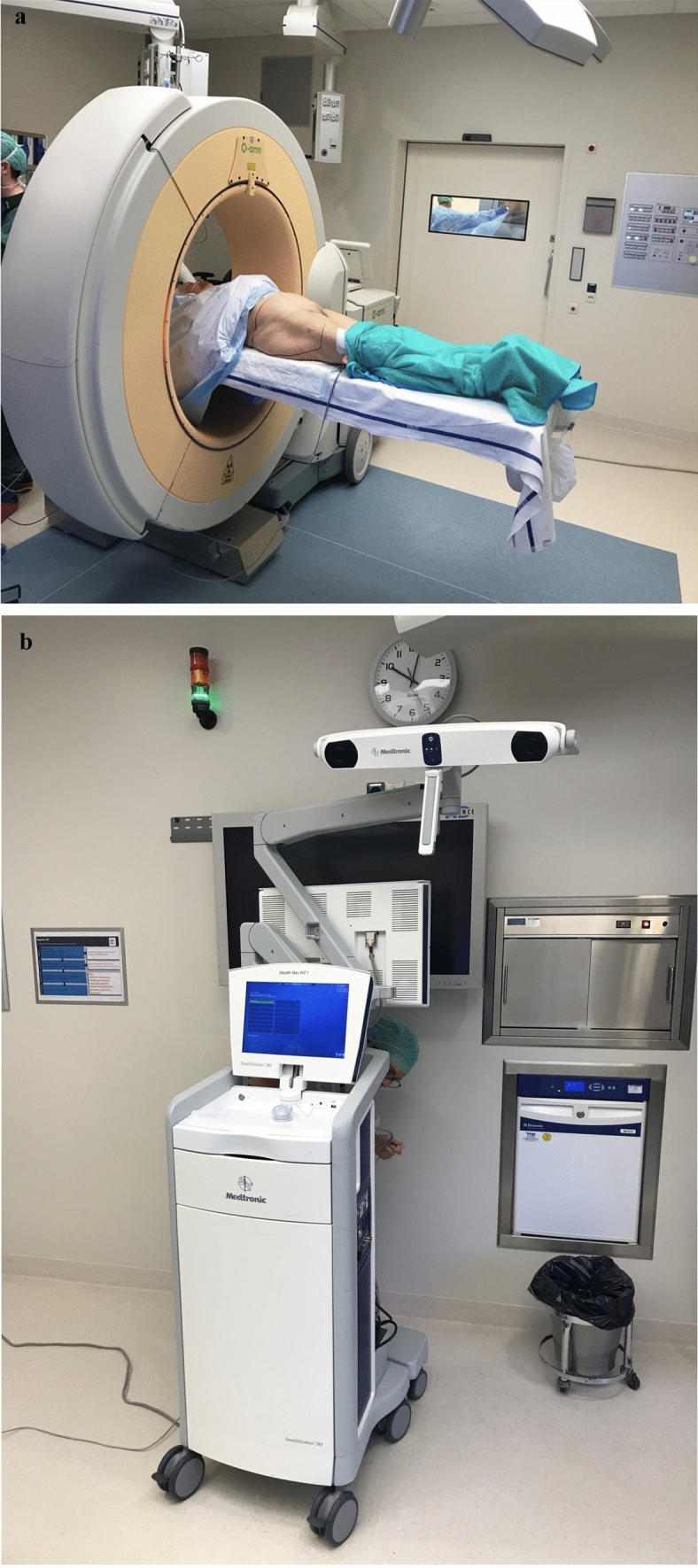
Standard surgical setup for posterior and anterior 3D-navigated pelvic screw fixation. **A **Positioning of the O-arm™ Imaging System (Medtronic, USA) prior to sterile draping, **B** StealthStation™ S8S (Medtronic, USA) positioned at the foot of the patient’s bed

**Fig. 2:**
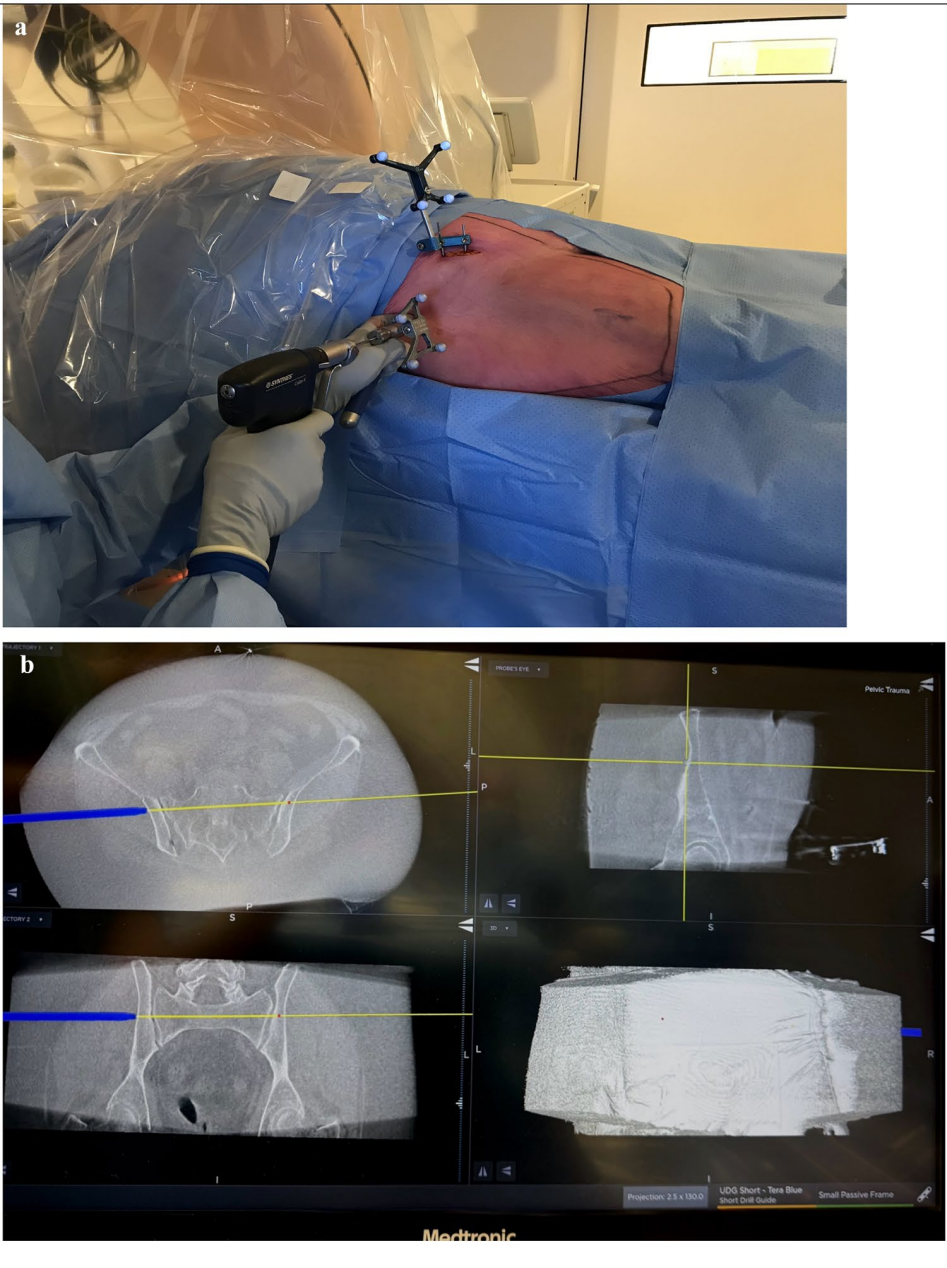
Standard surgical setup for posterior and anterior 3D-navigated pelvic screw fixation. **A** Reference frame attached to two percutaneously inserted K-wires in the patient’s iliac crest, shown in conjunction with the corresponding navigated drilling guide (Medtronic, USA), **B** StealthStation™ S8S (Medtronic, USA) navigation interface illustrating the projected drilling trajectories superimposed on the intraoperative O-arm recon scan. The display demonstrates multiplanar imaging (clockwise from the lower left quadrant: coronal, transverse, and sagittal planes) in addition to a three-dimensional reconstruction (lower right quadrant)

All included patients were operated on using a standardized surgical set-up. Patients were positioned supine on a radiolucent sacral bone foam positioning block and receiving general anaesthesia. Following sterile draping, the iliac crest was palpated, and a two-pin reference frame™ (Medtronic, USA) for the StealthStation™ S8S navigation system (Medtronic, USA) was percutaneously attached to the superior iliac crest (Figs. 1,2A). Three-dimensional images were generated using the O-arm™ Imaging System (Medtronic, USA) linked to the StealthStation™ S8S navigation system (Medtronic, USA), which determines the patient’s position and orientation via the reference frame™ (Medtronic, USA) (Fig. 2A). To minimize radiation exposure to the operating team, staff temporarily exited the operating room while a scout scan was obtained using the O-arm™ Imaging System, following the same imaging protocol for all patients. The intraoperative scan from the O-arm™ Imaging System (Medtronic, USA) was displayed in three planes: coronal, sagittal, and transverse, with an additional three-dimensional reconstruction (Fig. 2B). The navigated drill sleeve™ (Medtronic, USA) was calibrated and used to plan skin incisions, with the projected trajectories overlaid on the scout scan to mark the incision site (Fig. 2B).

A 3-cm skin incision was made, and blunt dissection down to the outer cortex of the ilium was performed. The calibrated navigated drill sleeve was inserted, and the K-wire was positioned through the sleeve. The projected trajectory was continuously monitored in three dimensions using the StealthStation™, which provides real-time feedback on the previously obtained scout scan. After placement of the navigated K-wire, a control run was performed using the O-arm to confirm correct positioning and rule out neuroforaminal perforation. If positioning was uncertain, the K-wire was removed and replaced, followed by another control scan. Once satisfactory placement within the correct bone corridor was confirmed, the screw trajectory was pre-drilled and a fully threaded 6.5 mm screw of the appropriated length including a washer was inserted over the K-wire.

Additional fractures of the anterior pelvic ring requiring fixation were addressed using the same incision and same procedure, targeting the superior ramus. Correct screw positioning of both the anterior and posterior screws was verified on a final O-arm scan if necessary.

### Ethics statement

The included patients represent a subset of a previously established database (Reference number 2020–125). The study was approved by the hospital’s board, and the medical ethics committee waived the requirement for informed consent for participation due to the observational nature of the research. Informed consent was obtained from all individuals depicted for the use of photographs in this publication. All data were handled in accordance with institutional guidelines and the principles of the Declaration of Helsinki.

### Statistical analysis

All data were analysed using IBM SPSS Statistics version 30. Continuous baseline characteristics and outcome measures were summarized using descriptive statistics (mean ± standard deviation or median with interquartile range, as appropriate). Categorical variables were presented as frequencies and percentages.

## Results

### Baseline

Of the 184 patients who underwent any form of pelvic fracture surgery in our centre, 90 patients received 3D-navigated screw fixation between 2019 and 2022 and were included in the study. In total, 180 posterior screws were placed: 120 transsacral-transiliac screws (TSTIS) and 60 sacro-iliac (SI) screws. Twenty-eight pubic ramus screws were placed. Five anterior screws were placed retrograde, and the remaining 23 screws were placed antegrade. The median operation time was 40.0 (8–133) minutes. Patient receiving non-percutaneous surgery or open reduction and internal fixation were excluded for this study. The mean radiation effective dose of all procedures was 24.4 mSV with a corresponding DLP of 4.71 Gy·cm^2^. The radiation exposure was primarily attributable to the intraoperative O-arm acquisitions, which typically consisted of two runs: an initial scout scan and a subsequent scan to confirm the position of the K-wires. In cases where K-wires required repositioning due to unsatisfactory placements, an additional scan was performed.

The majority (75%) of the patients were female with a mean age of 63.6 (± 22.4) years. Fall from standing (43, 47.8%) followed by fall from height (21,23.3%) were the most common trauma mechanism (Table 1).

**Table 1:** Patient characteristics

	All patients *n*=90
Age; mean (standard deviation)	63.6 (22.4)
Female; *n* (%)	68 (75.6)
High energy trauma; *n* (%)	38 (42.2)
Trauma mechanism; *n* (%)	
Fall from standing	43 (47.8)
Fall from height	21 (23.3)
Traffic accident	15 (16.7)
Other	3 (3.3)
Unknown trauma mechanism	8 (8.9)
**Fracture classification Youngs and Burgess; n (%)**	
APC-2	1 (1.1)
APC-3	2 (2.2)
LC-1	46 (51.1)
LC-2	5 (5.6)
LC-3	21 (23.3)
VS	7 (7.8)
Combined	2 (2.2)
Isolated sacral fracture	6 (6.7)
**Fracture classification Rommens; n (%)**	
FFP IIa	3 (3.3)
FFP IIb	21 (23.3)
FFP IIIa	1 (1.1)
FFP IIIb	1 (1.1)
FFP IIIc	7 (7.8)
FFP IVb	6 (6.7)
FFP IVc	9 (10.0)

Patients were admitted in the hospital for a median period of 7.5 days (1–66). Prolonged hospital stays were primarily related to difficulties in arranging discharge to rehabilitation facilities or home, rather than to medical complications. Surgery was performed at a median of 2.0 days (0–14) after admission, and after a median of 5.0 days (1–63), patients were discharged (Table 2).

**Table 2:** Surgical Outcomes

	All patients *n*=90
Hospital admission in days; median (range)	7.5 (1–66)
Surgery after admission in days; median (range)	2.0 (0–14)
Discharge after surgery in days; median(range)	5.0 (1–63)
**Delayed Surgery; n (%)**	22 (24.4)
Delayed presentation	13
Initial conservative period at home	5
Referral from another clinic	4
Delayed surgery after days; median (range)	45.0 (6–338)
**Technical complications; n (%)**	2 (2.7)
Screw misplacement	2
Perforation of neuroforamina	0
**Surgical complications; n (%)**	7 (7.8)
Superficial surgical site infection	2
Deep surgical site Infection	1
Non-union	4
**Neurological complications; n (%)**	7 (7.8)
Loss of sensation	1
Decreased motoric function	2
Persistent neuropathic pain	4
**Material related complications; n (%)**	14 (16.7)
Screw breakage of pubic ramus screw	1
Screw loosening SI screws	6
Screw loosening pubic ramus screws	2
Screw removal due to pain	5

### Post-operative outcomes

Screw misplacement occurred in 2 of 90 patients (2.7%). In both cases, the transiliac-transsacral screw showed a minimal cortical breach of the anterior cortex of the ipsilateral sacral ala. Importantly, no neuroforaminal breach or iatrogenic injury to neurovascular structures was observed in any patient (Table 2). Three patients were diagnosed with surgical site infection (SSI), all sustained a high-energy trauma. None of these fractures were open, nor had they undergone prior temporary external fixation. In two patients, the superficial infections occurred at the insertion site of the posterior TSTI screw and could be successfully treated with oral antibiotics (flucloxacillin) for 7 days. In one patient suffering from a Morel-Lavallée lesion on the right thigh after a high-energy trauma, a deep infection occurred at the insertion’ site of the posterior TSTI screws. Multiple operative debridements were needed, and the patient was treated with intravenous antibiotics for an extensive period; however, no removal of internal fixation material was needed. Material-related complications required screw removal in 14 patients (16.7%). The majority of removals were due to pain related to the implants, followed by screw loosening of the SI screws in six patients and backing out of pubic ramus screw in two patients. In addition, one patient required removal of a pubic ramus screw due to breakage (Table 2).

Seven (7.8%) patients reported neurological complications (Table 2). All these patients were referred to the neurologist for further examination. After careful evaluation by the neurologist, the neurological complaints were attributed to the traumatic injuries and not to pre-existing conditions or the surgery/screw placement. Retrospectively, the patient also acknowledged that these complaints had been present after the accident and prior to surgery which could be confirmed by the documentation in the patient files.

## Discussion

In this study, we investigated the safety of 3D-navigated screw fixation in 90 patients with pelvic ring fractures resulting from low- or high-energy trauma. A total of 180 posterior screws were placed, including 120 TSTI screws and 60 SI screws, along with 28 pubic rami screws. Our study demonstrated a very low rate of screw misplacement of 2.7%, limited to minor perforation of the anterior cortex of the sacral ala on the ipsilateral side, without any neuroforaminal perforation or neurological injury. These results align with the literature reporting 0–10% misplacement using 3D navigation and represents a clear improvement compared to the reported incidence of 15–20% described with conventional 2D fluoroscopy. [2, 16–19]. Similarly, recent studies by Gilli et al., Zwingmann et al., and Richter et al [20–22]. reported slightly lower accuracy rates of 83%, 81%, and 85%, respectively, for screw placement without cortical or neuroforaminal perforation, compared to 96.3% in our series. We believe that this superior accuracy of 3D-navigated screw fixation in pelvic ring fractures is largely attributable to the intraoperative real-time visualization of the osseous sacral corridors and the feedback on the projected screw trajectories provided to the surgeon. This is particularly valuable in complex cases, such as those involving sacral dysmorphism in S1 (present in 41% of the patients), and is supported by a recent review including 3D screw navigation in such anatomically challenging cases [18].

The mean effective radiation dose per patient in our study was 24.4 mSv originating from the intraoperative O-arm runs with a dose length product (DLP) of 4.71 Gy·cm^2^. These values are in line with previously reported data on spine and pelvic screw fixation using 3D navigation [23–26]. The major limitation in comparing reported radiation doses across studies is the considerable variability which is influenced by the patient characteristic (e.g. body mass index), differences in scan protocol and in reporting of radiation units, making direct comparisons difficult. Importantly, the radiation exposure to the surgical teams was minimized by temporarily leaving the operating room during scanning, in accordance with the standard radiation safety protocols. This represents a key advantage over 2D fluoroscopy-based navigation where spot or continuous imaging is required during K-wire a screw insertion and the surgical team is directly exposed to the radiation. Consistent with previous reports, 3D navigation reduces radiation exposure to the surgical team, as they can leave the operation room during scanning [19, 27, 28]. For the patients, however, whether overall radiation exposure is reduced remains controversial; doses appear higher with 3D navigation but remain within acceptable limits when compared to 2D fluoroscopy. The implementation of low-dose CT protocols into the 3D navigation workflows has the potential to substantially reduce patient exposure without compromising image quality. Ultra-low-dose pelvic CT with thin filtered scanning can achieve effective doses as low as 0.38 mSV (approximately six times lower than standard pelvic CT scans) and overall dose reduction of 55–60% reported in recent studies [29, 30].

Surgical site infections (SSIs) occurred in three patients (3.3%), all following high-energy trauma closed pelvic ring fractures. Two patients experienced superficial infections which were successfully managed with oral antibiotics. One patient with a Morel-Lavalée lesion developed a deep infection of the posterior TSTI screw site, requiring multiple operative debridements and prolonged intravenous antibiotic therapy. Importantly, no hardware removal was required because of SSI. SSI infection in our cohort is low, and similar to the reports in the literature, SSIs appear more related to the severity of the trauma and associated soft tissue injury rather than the navigated procedure itself [31–33]. 3D-navigated screw placement using the O-arm™ does not increase the risk of post-operative infection compared to conventional techniques [16, 18]. Although the surgical team briefly leaves the operating room during scanning and thus exits the laminar flow zone above the sterile field, the influence on SSI appears negligible.

Although no hardware removal was required due to infection, hardware removal was necessary in 14 (16.7%) of the patients. These events were not related to the navigation technique or screw accuracy, but rather to biomechanical factors, such as symptoms related to the osteosynthesis (*n*=5) or loss of screw purchase resulting in backing out of the SI screws (*n*=6). Seven patients (7.8%) reported neurological complaints, and all were evaluated by a neurologist. For all patients, the complaints were attributed to the initial traumatic injuries rather than the surgical procedure or screw placement. Notably, neither of the two patients with screw misplacement experienced neurological symptoms, and their hardware was retained. These findings highlight that patient-specific factors, such as fracture biomechanics, fracture pattern, and trauma mechanism, appear to be more relevant causes of post-operative complaints than the navigation technique.

While our results are encouraging, limitations must be acknowledged. The single-centre design and the modest sample size of 90 patients may affect the generalizability of the findings. Because 3D screw navigation in pelvic fractures is the standard of care in our institution, comparative data with 2D fluoroscopy were not available. Long-term outcomes, including functional aspects and quality of life as well as cost-effectiveness, were not addressed, as our focus was on procedural safety and accuracy. The efficacy and safety of this technique also depend on a dedicated, well-trained surgical team. We believe that the high accuracy, relative short operation time of 40 min, and the low SSI percentages achieved in our series are the result the expertise of our surgical team. Nonetheless, evidence indicates that 3D navigation is generally more accessible for novice orthopaedic surgeons and has a relatively short learning curve of 15–35 cases, with near-perfect accuracy achievable even in less experienced hands [2, 34]. In contrast, estimates for 2D fluoroscopy screw navigations suggest that 40–50 cases are required for consistent accuracy, with misplacement rates up to 20% even among experienced surgeons [2, 35, 36]. Whether the safety profile of traditional 2D fluoroscopy navigation can match 3D-navigated screw placement in pelvic fractures remains uncertain. Acceptable misplacement rates with 2D fluoroscopy appear to be achievable primarily in high-volume pelvic trauma centres. Our hospital, one of ten Level 1 trauma centres in the Netherlands and a tertiary referral centre for pelvic fractures, does not reach the annual pelvic ring fracture volumes seen in the major US centres. This reflects a common situation in the Netherlands, where dense population combined with a relatively high number of Level 1 trauma centres results in less centralization and lower per-centre case volumes compared to the USA [18, 37] Consequently, Dutch centres generally treat fewer pelvic fractures annually, whereas in USA, 2D fluoroscopy remains the standard of care.[38–40].

Given our findings and current evidence, we advocate for the routine implementation of 3D navigation in percutaneous screw fixation of pelvic ring fractures, particularly in medium-volume centres such as those in the Netherlands.

This technology not only improves the accuracy and safety of screw placement in pelvic ring fractures but also simplifies the procedure, enhances surgical training, and is particularly beneficial in anatomically complex cases, including those with sacral dysmorphism.

## Conclusions

Our study demonstrates that 3D-navigated screw fixation for pelvic ring fractures is safe and effective, with low screw misplacement. Although occasional perforation of the anterior cortex may occur, these events did not result in neurological injury and are minor compared to the higher misplacement and neurovascular risk associated with conventional 2D fluoroscopic screw navigation. Based on these findings, we recommend adopting 3D navigation for percutaneous screw placement in pelvic ring fractures whenever available. This approach provides an accurate, reproducible, and safe method, particularly in anatomically complex regions of the sacrum.

## Data Availability

The datasets used and/or analysed during the current study are available from the corresponding author on reasonable request.
